# Infectious disease outbreaks among forcibly displaced persons: an analysis of ProMED reports 1996–2016

**DOI:** 10.1186/s13031-020-00295-9

**Published:** 2020-07-22

**Authors:** Angel N. Desai, John W. Ramatowski, Nina Marano, Lawrence C. Madoff, Britta Lassmann

**Affiliations:** 1 International Society for Infectious Diseases, Brookline, MA USA; 2 Brigham & Women’s Hospital, Division of Infectious Disease, Boston, MA USA; 3 Centers for Disease Control and Prevention, Atlanta, GA USA; 4 University of Massachusetts Medical School, Worcester, MA USA

**Keywords:** Surveillance, Forced displacement, Outbreak, Infectious disease, ProMED

## Abstract

**Background:**

The United Nations Refugee Agency (UNHCR) estimates the number of forcibly displaced people increased from 22.7 million people in 1996 to 67.7 million people in 2016. Human mobility is associated with the introduction of infectious disease pathogens. The aim of this study was to describe the range of pathogens in forcibly displaced populations over time using an informal event monitoring system.

**Methods:**

We conducted a retrospective analysis of ProMED, a digital disease monitoring system, to identify reports of outbreak events involving forcibly displaced populations between 1996 and 2016. Number of outbreak events per year was tabulated. Each record was assessed to determine outbreak location, pathogen, origin of persons implicated in the outbreak, and suspected versus confirmed case counts.

**Results:**

One hundred twenty-eight independent outbreak events involving forcibly displaced populations were identified. Over 840,000 confirmed or suspected cases of infectious diseases such as measles, cholera, cutaneous leishmaniasis, dengue, and others were reported in 48 destination countries/territories. The average rate of outbreak events concerning forcibly displaced persons per total number of reports published on ProMED per year increased over time. The majority of outbreak events (63%) were due to acquisition of disease in the destination country.

**Conclusion:**

This study found that reports of outbreak events involving forcibly displaced populations have increased in ProMED. The events and outbreaks detected in this retrospective review underscore the importance of capturing displaced populations in surveillance systems for rapid detection and response.

## Background

Human mobility has long been associated with the introduction of infectious disease pathogens, transmission, and propagation globally. Severe acute respiratory syndrome (SARS), Middle East respiratory syndrome (MERS), and Zika virus are contemporary examples of outbreaks that underscore the role of mobility, travel, and migration in the spread of infectious diseases [1–3]. Conflict, insecurity, fear of persecution, natural disasters, and environmental pressures have contributed to population displacement. The United Nations High Commissioner for Refugees (UNHCR) estimated that in 1996, 22.7 million people were classified as forcibly displaced people worldwide [4]. By 2016, this number had increased to 67.7 million people [4].

Infectious disease surveillance among forcibly displaced populations is challenging. Humanitarian crises disrupt local healthcare systems, preventing implementation of routine public health safeguards [5]. Forcibly displaced populations are not always captured in national surveillance systems because of challenges accessing health services that may be available to national or insured individuals only, and in the case of refugees and undocumented migrants, uncertain political status within their country of asylum. Disease surveillance methodologies for displaced populations vary; some recent studies have assessed outbreaks using formal, laboratory-based, and syndromic surveillance to identify events in specific locations and over defined time periods [6–9]. For example, the UNHCR Health Information System (HIS) is a syndromic surveillance system that tracks morbidity due to a wide variety of communicable diseases in UNHCR refugee camps. HIS has been configured so that weekly reports of these syndromes can be aggregated and reviewed with an alert threshold for outbreak detection set according to the disease of concern [10]. The principle burden of infectious diseases in forcibly displaced populations is due to endemic diseases such as acute respiratory infections, endemic diarrhea, measles, and malaria [11]. A comprehensive analysis regarding the occurrence and extent of outbreaks of infectious diseases in forcibly displaced populations over time however, is lacking in the published literature.

Informal, digital monitoring and reporting tools may be useful adjuncts to traditional disease surveillance in these scenarios, as they require fewer resources and often encounter fewer bureaucratic barriers [12–15]. In addition, given that population displacement is ongoing and dynamic, informal reporting offers a complementary mechanism to highlight cross-border infectious disease developments over longer periods of time. We analyzed infectious disease outbreak reports published on ProMED to investigate the pace and extent of infectious disease outbreaks in forcibly displaced populations over the study period, as well as highlight global pathogen-specific findings and trends.

## Methods

### Data source

Informal monitoring and reporting systems rely on local media, professional networks, and on-the-ground experts to highlight emerging infectious diseases and outbreaks in near real time. Examples include the Program for Monitoring Emerging Diseases (ProMED) published by the International Society for Infectious Diseases; HealthMap; and the Global Public Health Intelligence Network (GPHIN) [13].

ProMED, a program of the International Society for Infectious Diseases, is a digital monitoring and reporting system for infectious diseases. ProMED was established in 1994, and allows human and animal health practitioners, public health professionals, and concerned members of the public to submit information regarding potential infectious disease outbreak events [16]. Media reports regarding infectious disease outbreaks have been mined using automated machine web crawling with manual curation since 2007 through collaboration with HealthMap [17]. Both formal and informal surveillance reports are submitted to ProMED, and a network of subject matter experts with knowledge of regional infrastructure and infectious diseases screen all reports. Subject matter experts comment on each report, adding context to a local outbreak when relevant. Once a report has been reviewed and edited, it is posted to the global ProMED network, where it is freely accessible and reaches more than 90,000 subscribers and followers. ProMED focuses its reporting on emerging and re-emerging outbreaks defined by the World Health Organization as “the occurrence of cases of disease in excess of what would normally be expected in a defined community, geographical area, or season [18]”. Chronic infectious diseases such as tuberculosis, chronic viral hepatitis, human immunodeficiency virus (HIV), and endemic infectious diseases are not routinely reported. ProMED reports have been validated by several other studies investigating emerging infectious diseases trends and outbreaks and have been shown to be a rapid and valuable system to obtain information about emerging outbreaks [19–25].

### Methodology

**Fig. 1:**
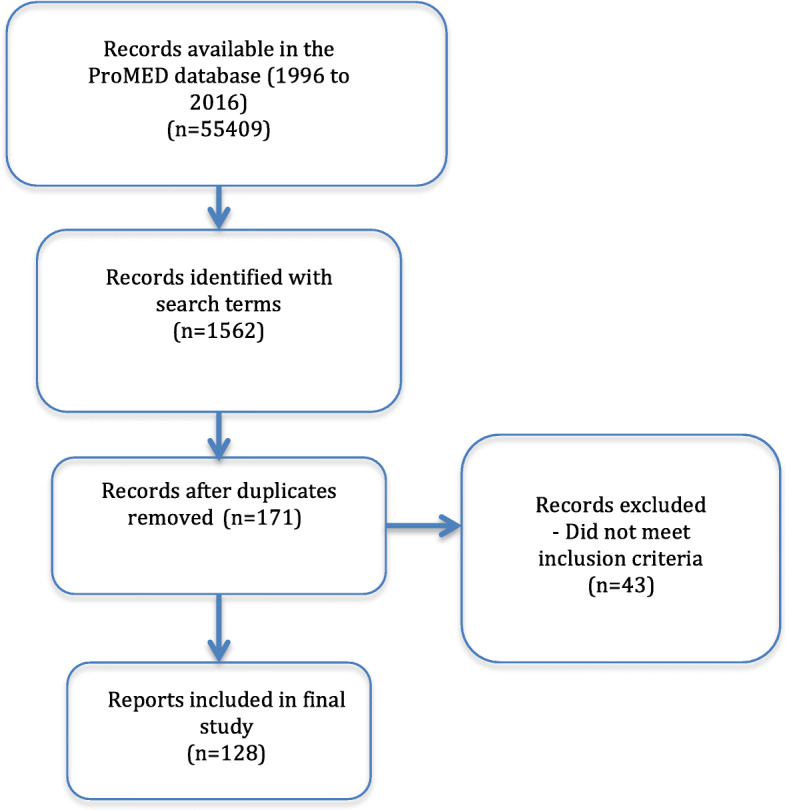
Flow Chart of search strategy

In this retrospective analysis, we focused on outbreak events among forcibly displaced populations reported in ProMED from 1996 to 2016. In formulating search terms for the inclusion criteria, we used UNHCR definitions of forcibly displaced populations as those displaced involuntarily by persecution, conflict, violence, natural disasters, or human rights violations [4]. This includes refugees, asylum seekers, and internally displaced persons (IDP). As a result, the ProMED archive was queried for the following search terms and root words: “refugee(s)” OR “asylum seeker(s)” OR “displaced”. Exclusion criteria included reports indicating voluntary population movement for economic benefit or family reunification, and reports that referred to the same outbreak. For example, in several instances one outbreak event was detailed by multiple reports in the ProMED archive, tracking the development of an infectious disease outbreak over time. As an example, a cholera outbreak following the 2010 Haitian earthquake was captured in ProMED with over 20 reports over 1 year. To ensure only unique outbreak events were assessed, the last report in a series with information pertinent to this study was used. Outbreaks in forcibly displaced persons from ProMED included reports in refugee camps as well as enclaves in cities or at borders. English-language reports from the ProMED global network were included in this analysis. Of the total 55,409 records in the ProMED database, 1562 returned with one of the identified search terms. Of these, 171 remained after removal of duplicates, and an additional 43 were removed as not meeting the inclusion criteria based on the pre-specified definition of forcibly displaced populations (Fig. 1). One hundred twenty-eight reports describing unique outbreak events were ultimately retained. Each record was manually reviewed to extract report date, location of outbreak, origin of groups associated with the outbreak event, pathogen implicated, and case counts (Fig. 1). Point of disease acquisition and, if applicable, reason for vaccination interruption were extracted from each report.

Three independent investigators thoroughly reviewed each report to ensure adherence to the criteria as outlined above. In some instances, case numbers reported in ProMED outbreak reports were rounded to the nearest 1000 or reported as “more than” a specific number, e.g. more than 100 cases were reported. Case numbers reported in this analysis therefore represent approximate numbers.

The rates of outbreak events involving forcibly displaced populations per total number of ProMED reports published each year were calculated. Rates of outbreak events among displaced populations per total number of ProMED reports per year were calculated to ensure that any variations observed were not secondary to changes in the number of ProMED reports published, and were compared during two time periods (1996–2002 and 2010 to 2016) using a two-sample t-test assuming unequal variance. *P* < 0.05 was considered statistically significant. Microsoft Excel and Stata (StataCorp. 2009. Stata Statistical Software: Release 11. College Station, TX: StataCorp LP) were used for statistical analysis.

## Results

**Fig. 2:**
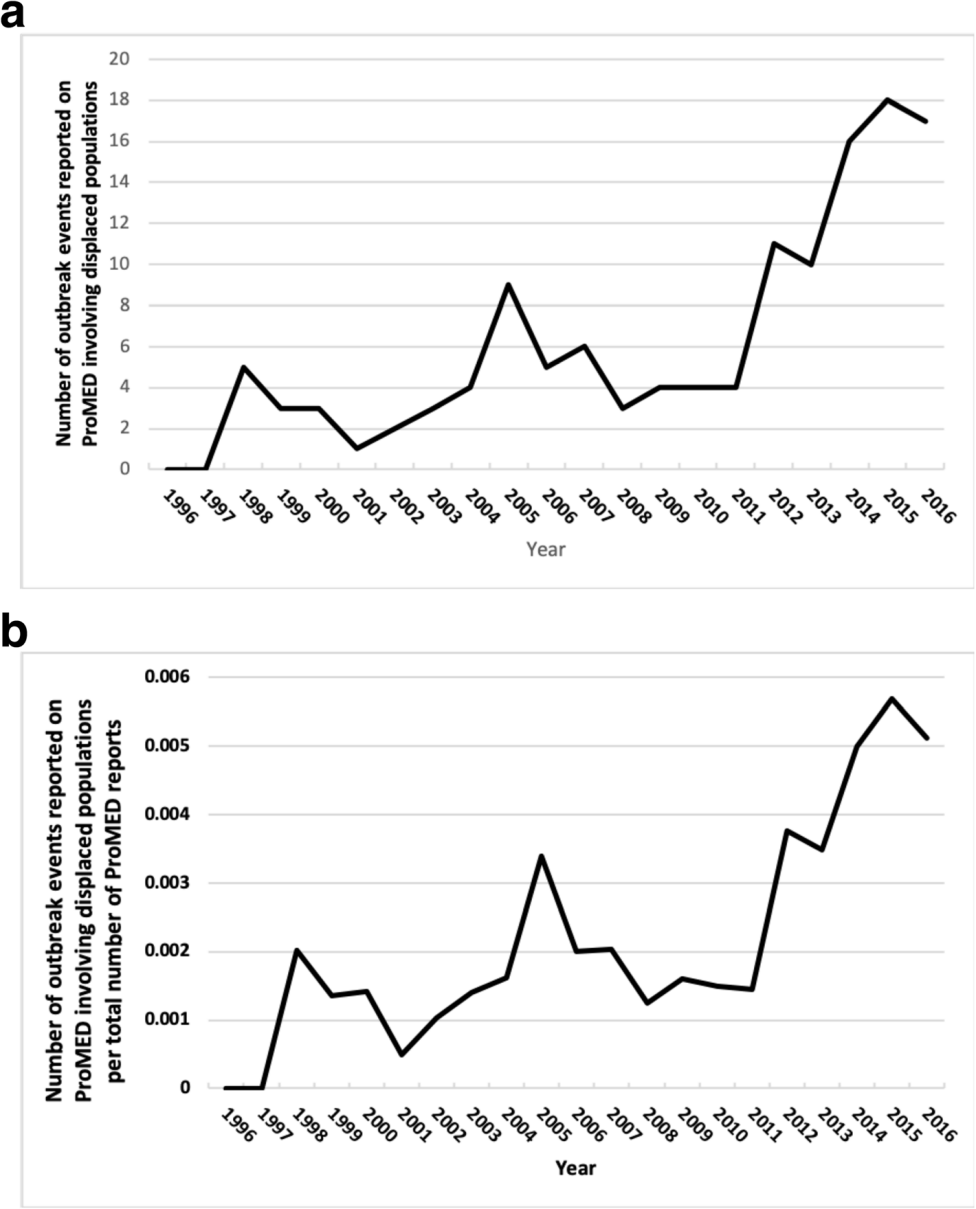
**a** Number of outbreak events involving displaced populations reported on ProMED from 1996 to 2016. **b** Number of ProMED outbreak events involving displaced populations per total number of ProMED reports from 1996 to 2016

Between 1996 and 2016, a total of 128 unique outbreak events involving forcibly displaced populations were posted on the ProMED network. The mean incidence of outbreak events involving forcibly displaced populations increased from an average of 2.3 events per year during 1996 to 2002 to 5.7 during 2003 to 2009 to 11.4 events per year during 2010 to 2016, an overall increase of 404% when comparing the first time period (1996 to 2002) to the last time period (2010 to 2016) (*p* < 0.05). Over the same periods, the average rate of outbreak events per total number of reports published on ProMED each year increased by 322% (*p* < 0.05). Outbreak events reported on ProMED and outbreak events involving displaced populations reported on ProMED per total number of ProMED reports from 1996 to 2016 are shown in Fig. 2a and b.

**Fig. 3:**
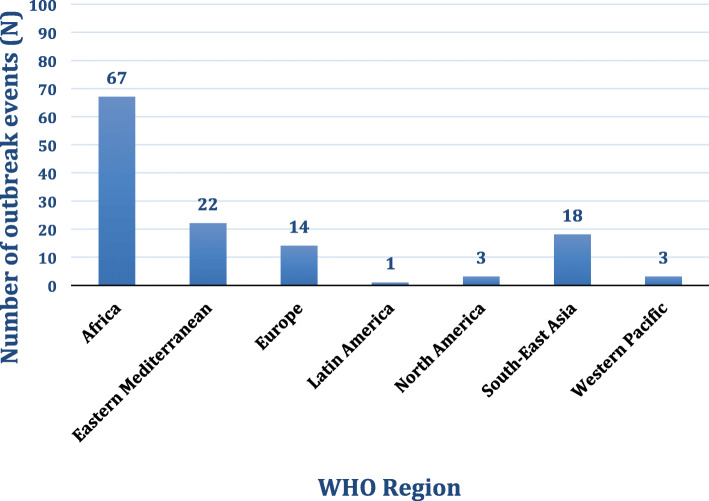
Infectious disease outbreak events in displaced populations by region, 1996–2016. For the purpose of this analysis, WHO Americas region was divided into North America and Latin America. *N* = 128

Over the 20-year study, outbreak events were reported in 48 destination countries/territories as detailed in Supplementary Table 1. Results were categorized by WHO region, and then by countries where relevant. Figure 3 demonstrates the distribution of individual outbreak events among WHO regions. For the purpose of this analysis, WHO Americas region was divided into North America and Latin America. The majority of outbreaks over the study period involving forcibly displaced populations were reported in Africa (52%; 67 reports), followed by Eastern Mediterranean (17%, 22) and Southeast Asia (14%, 18). The study population was primarily identified as refugees (60.9%, 70), followed by internally displaced persons (29.6%, 38) and asylum seekers (9.3%, 12) per UNHCR definitions. While study populations were often reported independently in one of these categories, we were unable to definitively determine in all reports if the affected population included a mix of refugees, IDPs, and asylum seekers.

Table 1 demonstrates all reported pathogens, count of outbreaks, and approximate suspected and confirmed case counts by WHO region. Supplementary Tables 1 and 2 provide additional details on outbreak location and approximate case counts. When a pathogen was not explicitly identified, “syndrome” as indicated in the corresponding ProMED report was used. In sum, more than 840,000 confirmed or suspected cases of infectious diseases were identified over the study period. Forty-one unique pathogens or syndromes were represented in this study, demonstrating a wide range of infectious diseases. While the majority of reported cases were due to a large-scale cholera outbreak among internally displaced people (IDP) affected by the 2010 Haiti earthquake (> 520,000 cases) and a large-scale epidemic typhus outbreak in Burundi in 1998 (100,000 cases), it is notable that within this dataset, a wide variety of other pathogens were seen. Outbreak events due to cholera (*n* = 34 outbreak events), cutaneous leishmaniasis (*n* = 6), dengue (*n* = 5), hepatitis E (*n* = 8), measles (*n* = 21) and poliomyelitis (*n* = 7) were highlighted as the most common disease outbreaks afflicting displaced populations. Overall, most individual cholera outbreaks (*n* = 23 outbreak events) occurred in the WHO Africa region over the study period.

**Table 1:** Count of outbreaks (approximate cumulative suspected and confirmed case counts) reported in displaced persons, by WHO region, 1996-2016. *N* = 128 (846319)

Reported Pathogen or Syndrome								
	Location of Outbreak by WHO Region							
	Africa	Eastern Mediterranean	Europe	Latin America^**a**^	North America^**a**^	South-East Asia	Western Pacific	Total
Malaria-like disease	1 (20)							1(20)
Acute abdominal pain						1 (85)		1 (85)
Anthrax						1 (1500)		1 (1500)
Bacterial meningitis	1 (141)							1 (141)
Chickenpox						1 (12000)		1 (12000)
VZV^b^ & scabies	1 (1000)							1 (1000)
Cholera	23 (15882)	5 (2262)		1 (527000)		5 (576)		34 (545720)
Cutaneous diphtheria			1 (9)					1 (9)
Cutaneous leishmaniasis		6 (152676)						6 (152676)
Dengue		1 (185)				2 (905)	2 (23)	5 (1113)
Diarrhea, unspecified			1 (500)			2 (1500)		3 (2000)
Diphtheria, non-cutaneous		1 (130)						1 (130)
Ebola hemorrhagic fever	1 (119)							1 (119)
Ebola-suspected			1 (1)					1 (1)
Gastroenteritis						1 (100)		1 (100)
Hepatitis E	8 (15793)							8 (15793)
Lassa fever	1 (88)							1 (88)
Leprosy						1 (not reported)		1 (−)
Louse-borne relapsing fever			3 (18)					3 (18)
Malaria					1 (15)	1 (50)		2 (65)
Marburg virus	1 (5)							1 (5)
Measles	12 (1563)	1 (42)	6 (974)		2 (5)			21 (2584)
Meningococcal meningitis	1 (26)							1 (26)
Meningitis, unknown pathogen		1 (6)				1 (4)		2 (10)
Monkeypox	1 (150)							1 (150)
Poliomyelitis	5 (128)	2 (70)						7 (198)
Rift Valley fever	1 (80)							1 (80)
Salmonella		1 (237)						1 (237)
Scabies		1 (1)						1 (1)
*Shigella flexneri*	1 (1062)							1 (1062)
Skin rashes & cholera						1 (6500)		1 (6500)
Syphilis			1 (112)					1 (112)
Tuberculosis		1 (50)						1 (50)
Tetanus						1 (72)		1 (72)
Trypanosomiasis	1 (490)							1 (490)
Typhoid fever		2 (99)	1 (24)				1 (2)	4 (125)
Typhus (epidemic)	1 (100000)							1 (100000)
Suspected yellow fever	1 (55)							1 (55)
Unknown	2 (23)							2 (23)
Visceral leishmaniasis	1 (1800)							1 (1800)
Yellow fever	3 (161)							3 (161)
Total	67 (138586)	22 (155758)	14 (1638)	1 (527000)	3 (20)	18 (23292)	3 (25)	128 (846319)

^a^WHO region ‘Americas’ subdivided here into Latin America and North America in order to display data at a more granular level

^b^*VZV* varicella zoster virus

Of all pathogens or syndromes implicated in infectious disease outbreaks, more than 16,000 vaccine-preventable disease cases (VPD) were identified, encompassing 39 outbreaks in 20 countries over the course of the study. VPD in this study included measles, poliomyelitis, diphtheria, tetanus, pertussis, yellow fever, typhoid, and varicella. Other childhood diseases such as mumps and rubella were not reported in our data set. As cholera vaccines and their uptake were not widespread during the study period, cholera was not included in the VPD case counts. Meningococcal disease was also excluded, as etiology of meningitis was not always made explicit in ProMED reports. The largest number of outbreaks of VPD was noted in Kenya as the host country (*n* = 7). Six of these outbreaks were noted in forcibly displaced persons from Somalia, with four of those outbreaks due to poliomyelitis and the remaining two related to measles. Within the poliomyelitis outbreak cohort, there were 125 suspected or confirmed cases with no fatalities. One measles outbreak in Kenya was noted in displaced individuals from South Sudan. The remaining VPD outbreaks across regions were distributed as demonstrated in Table 2.

**Table 2:** Distribution of vaccine-preventable outbreaks in displaced persons by location of outbreak, 1996–2016. *N* = 39

Outbreak Location								
	Measles	Poliomyelitis	Tetanus	Typhoid	Diphtheria	Yellow Fever	Varicella	Total
Australia				1				1
Chad	1							1
Ethiopia	1							1
France	1							1
Georgia				1				1
Germany	3							3
Indonesia			1					1
Kenya	3	4						7
Kyrgyzstan	1							1
Lebanon		1						1
Macedonia	1							1
Nigeria	1	1					1	3
Pakistan		1			1			2
South Sudan	3							3
Sri Lanka							1	1
Sudan	1					3		4
Syria	1			2				3
Tanzania	1							1
Uganda	1							1
United States	2							2
Total	21	7	1	4	1	3	2	39

Origin and destination of populations and subsequent outbreak events were investigated in this analysis. On a country level, Kenya experienced the largest number of distinct outbreaks (*n* = 13) reported to ProMED, followed by Uganda (*n* = 12). Reports were manually reviewed for explicit indication of disease acquisition at the destination as opposed to importation from the location of origin or during transit. Of 128 reports, 63% (80) were due to local transmission in the destination country, 20% (25) were due to importation (acquired in the country of origin or during transit), and 18% (23) were unspecified based on manual review of the report. In cases of importation, 48% (12) events were attributed to incomplete vaccination of the displaced population. The reasons for interrupted vaccination when available, were extracted from reports, and cited as breakdown in local health infrastructure and mistrust of local medical care.

## Discussion

Global migration has reached unprecedented levels over the past decade. This study examined the relationship between forced migration and infectious disease outbreaks by using the ProMED database, an informal, digital disease monitoring and reporting system. It demonstrated a wide variety of infectious disease pathogens or syndromes affecting many countries and cross-border regions. Our findings show that the number of ProMED infectious disease outbreak reports concerning forcibly displaced populations has increased over the study period. Rates of outbreak events concerning forcibly displaced populations per total number of ProMED reports also increased; the observed changes were not solely caused by an increase in the total number of ProMED reports. The number of displaced persons estimated worldwide has steadily risen as the number of ProMED reports in this population has increased. During the study period, the average annual forcibly displaced persons’ population size increased from approximately 21 million between 1996 and 2002, to 28 million during 2003–2009, to 48 million from 2012 to 2016 [4]. A direct association between the increase in the number of ProMED reports and forcibly displaced persons’ population size could not be made in this study. Despite this increase in outbreak reports, outbreak-related case counts in forcibly displaced persons totaled only 846,319 compared to the millions of individuals who were forcibly displaced over the 20-year study period. In this dataset, disease outbreaks due to VPD were often due to the collapse of public health measures. It bears mentioning that the relatively low case counts may be due in part to incomplete data or underreporting, which remains a challenging issue in this population, even when considering the advantages of informal surveillance methodology. As innovation in the development and integration of additional data streams that can provide more granular information for informal surveillance systems become available, this limitation may be partially remedied in the future. Outbreaks in forcibly displaced persons from ProMED included reports in refugee camps as well as enclaves in cities or at borders, but limitations in outbreak surveillance occur when forcibly displaced persons are integrated with host populations. Given the time of our search, the beginning of large waves of migration from Syria and Iraq in 2011, South Sudan, Burundi, DR Congo, and the Central African Republic starting in 2015, and the Venezuelan crisis that began in 2014 were captured here but do not necessarily reflect the entirety of migration patterns and subsequent outbreaks.

The highest number of ProMED outbreaks was seen in Kenya. During the study period, Kenya was home to at least four of the largest refugee camps in the world, which may account for the numbers of outbreaks seen in that country over the course of this analysis [4]. Outbreak events in this study involved primarily local acquisition as opposed to importation. These findings are consistent with prior studies that have demonstrated low risks of imported acute infectious diseases on host country epidemiology, while crowding associated with temporary resettlement increases the risk of outbreaks among displaced residents [26, 27]. Of note, our results demonstrate that while the risks were low, cases of imported infectious diseases did occur in host countries. These cases appeared to be due in large part to collapsing health infrastructure in countries of origin. As demonstrated in our evaluation, nearly half of all imported infectious diseases were vaccine-preventable, with some reports noting that incomplete vaccination due to political conflict and subsequent deterioration of public health infrastructure was the cause of disease outbreaks. This may be explained in part, because securing high rates of vaccine coverage in countries of conflict is often difficult, and engaging forcibly displaced populations in host countries for this purpose can be equally challenging. Overall, a substantial number of outbreaks (30.4%, *n* = 39) in this study were related to VPD. Given the emphasis of ProMED on acute outbreaks, chronic infectious diseases are not reflected in our data. Recent data suggest that the prevalence of chronic infectious diseases such as viral hepatitis in forcibly displaced populations tends to depend on the epidemiology in their country of origin [28, 29].

Vigilant surveillance, adequate healthcare, immunization services, and infection control are critical to preventing morbidity and mortality in forcibly displaced persons. In this population, the principle burden of infectious diseases is due to endemic diseases such as acute respiratory infections, endemic diarrhea, measles, and malaria [11]. ProMED focuses its monitoring and reporting on emerging and re-emerging outbreaks as defined by the WHO [18]. In the context of endemic infectious diseases, ProMED reports unusual cases or the occurrence of cases of disease in excess of what would normally be expected in a defined community, geographical area, or season. In our dataset, outbreak events due to cholera, cutaneous leishmaniasis, dengue, hepatitis E, measles and poliomyelitis were highlighted as the most common disease outbreaks afflicting displaced populations. Public health investment and education concerning appropriate water, sanitation, and hygiene practices are pivotal steps to preempt diarrheal disease outbreaks in vulnerable populations, particularly in refugee camps [6]. The role of local and international agencies in supporting vaccination programs for refugees and internally displaced populations should be clearly delineated to capitalize on the operational strengths of each partner [30]. Our findings support a recent study that suggested that measles outbreaks among migrants in the European Union were due in part to sub-optimal vaccination coverage [31]. Enhanced cross-border surveillance with targeted screening and treatment of infectious diseases have been demonstrated to improve the health outcomes of forcibly displaced persons before resettlement and is recommended to be pursued in outbreak settings as well [32–35].

Our study methodology has several strengths. Disease reporting and monitoring systems such as ProMED may allow for the characterization of outbreak data that may be missed in official national surveillance reporting, given the precarious legal status of refugee and IDP populations. Similarly, ProMED is free of political constraints that might otherwise hinder reporting on these populations, allowing for unique data collection opportunities. In addition, subject matter experts review ProMED reports and often highlight specific information about special populations, case counts, or etiology of disease events within the report, allowing for further contextualization of each outbreak. Monitoring disease outbreaks in forcibly displaced populations is a complex endeavor. Some systems may focus only on temporary settlements, missing data from other settings. National surveillance methods may not report on internally displaced persons, limiting epidemiological discourse. While the dataset discussed in this paper does not provide a comprehensive overview of all outbreak-related trends in forcibly displaced persons worldwide, it does provide a longitudinal and global outlook on populations that can be difficult to characterize by virtue of their mobility and the limitations of current disease reporting tools.

This study has a few limitations. Only ProMED was used for this analysis, and this dataset does not provide a complete overview of all outbreaks among forcibly displaced persons over the study time period. The rationale driving an individual’s decision to migrate may be complex, and it is possible that some persons who were classified as forcibly displaced migrated for both voluntary and involuntary reasons. Given the nature of the ProMED dataset, some reports may have been misclassified, thereby overestimating or underestimating the number of reports referring to forcibly displaced persons for the purposes of this analysis. As discussed previously, the increase in the number of outbreak events related to forcibly displaced populations identified by this study might be the result of increased reports posted to ProMED. The use of automated mining for data on outbreaks in 2007 for example, may have increased the number of ProMED reports. Similarly, increasing Internet penetrance over time worldwide may have contributed to a steep rise in disease events. In order to mitigate these potential biases, we used total reports per year as the denominator in order to normalize the data across time periods. Countries with stronger informal reporting systems or more reliable Internet connections at baseline may be disproportionately represented in some of the reported outbreaks as well. Increased awareness of this population might also have contributed to increased reporting over time. Case counts and the identification of the last ProMED outbreak report in the series may have been affected by the terminology used in the body of the post and the search terms selected for this study. Diarrheal disease not otherwise specified was categorized independently of cholera. As a result, the occurrence of cholera in this study population may be underestimated, as cases of cholera may have been misclassified under diarrheal disease. Influenza-like illness causes considerable morbidity in this population but was not reported in the ProMED dataset [36]. Chronic infectious diseases and non-communicable diseases were not considered here, although morbidity associated with non-communicable diseases warrants further investigation. Finally, it is important to note that the data presented here represent a static overview of outbreaks over the study period. Migration patterns change over time in the setting of new conflicts or economic upheaval, inexorably affecting infectious disease epidemiology. Our analysis focuses on overall trends as opposed to potentially changing events over the study period.

## Conclusions

The response to disease outbreaks in forcibly displaced populations can be enhanced through clear lines of communication, and with varied surveillance systems working together to enable prompt detection and isolation of emerging threats. This is the first study to our knowledge that has used informal disease monitoring to provide a global and long-term description of infectious disease outbreaks among forcibly displaced persons. While the focus of this analysis used ProMED data, future studies should include additional informal disease monitoring and reporting systems to elucidate comprehensive, longitudinal outbreak trends among forcibly displaced populations. The outbreak events documented in this study demonstrate the need for timely and accurate infectious disease monitoring and reporting tools in humanitarian crises.

## Supplementary information

**Additional file 1: Supplementary Table 1.** Outbreak report counts by country/territory as reported by ProMED. **Supplementary Table 2.** Cases (suspected and confirmed) in displaced persons reported in ProMED by year^†^.

## Data Availability

All reports are publically available at www.promedmail.org. The datasets used and/or analyzed during the current study are available from the corresponding author on reasonable request.
