# Supplementary material for: Infectious disease outbreaks among forcibly displaced persons: an analysis of ProMED reports 1996–2016
Source: Confl Health. 2020 Jul 22;14:49. doi: 10.1186/s13031-020-00295-9 (PMC7374653; doi:10.1186/s13031-020-00295-9)
Supplement: Supplementary file 1 — Additional file 1: Supplementary Table 1. Outbreak report counts by country/territory as reported by ProMED. Supplementary Table 2. Cases (suspected and confirmed) in displaced persons reported in ProMED by year†. [file 13031_2020_295_MOESM1_ESM.docx]

**Supplementary Table 1. Outbreak report counts by country/territory as reported by ProMED**

| Country | Reports (Count) |
| --- | --- |
|  |  |
| Afghanistan | 1 |
| Australia | 2 |
| Burundi | 1 |
| Cameroon | 1 |
| Canada | 1 |
| Chad | 5 |
| DRC | 4 |
| East Timor | 1 |
| Ethiopia | 2 |
| France | 1 |
| Georgia | 1 |
| Germany | 4 |
| Germany, Sweden, and Denmark* | 1 |
| Ghana | 1 |
| Haiti and Dominican Republic* | 1 |
| India | 1 |
| Indonesia | 3 |
| Iran | 1 |
| Iraq | 2 |
| Israel | 1 |
| Italy | 1 |
| Jordan | 2 |
| Kenya | 13 |
| Kyrgyzstan | 1 |
| Lebanon | 2 |
| Republic of North Macedonia | 1 |
| Myanmar | 2 |
| Nauru | 1 |
| Netherlands | 1 |
| Niger | 3 |
| Nigeria | 4 |
| Pakistan | 5 |
| Republic of Congo | 1 |
| Sierra Leone | 1 |
| Somalia | 1 |
| South Sudan | 6 |
| Sri Lanka | 3 |
| Sudan | 10 |
| Switzerland | 1 |
| Syria | 6 |
| Tanzania | 3 |
| Thailand | 8 |
| Uganda | 12 |
| United States | 2 |
| West Bank | 1 |
| Yemen | 2 |
| **Total** | **128** |

*Some outbreaks involved cross-border transmission. All affected countries for these outbreaks are listed together in the table.

**Supplementary Table 2. Cases (suspected and confirmed) in displaced persons reported in ProMED by year^†^**

| **Year** | **Case Count (Approximate suspected and confirmed)** |
| --- | --- |
| 1996 | 0 |
| 1997 | 0 |
| 1998 | 102185 |
| 1999 | 798 |
| 2000 | 634 |
| 2001 | 490 |
| 2002 | 181 |
| 2003 | 112 |
| 2004 | 3945 |
| 2005 | 7541 |
| 2006 | 597 |
| 2007 | 1100 |
| 2008 | 1609 |
| 2009 | 13080 |
| 2010 | 1725 |
| 2011 | 564 |
| 2012 | 532141 |
| 2013 | 12878 |
| 2014 | 4092 |
| 2015 | 9979 |
| 2016 | 152668 |
| **Total** | 846319 |

^†^Cases reported in ProMED are subject to significant variability and may not capture the entirety of case definitions or deaths in individual outbreaks.
